# Advancements in Wearable Digital Health Technology: A Review of Epilepsy Management

**DOI:** 10.7759/cureus.57037

**Published:** 2024-03-27

**Authors:** Abhinav Ahuja, Sachin Agrawal, Sourya Acharya, Nitesh Batra, Varun Daiya

**Affiliations:** 1 Medicine, Jawaharlal Nehru Medical College, Datta Meghe Institute of Higher Education & Research, Wardha, IND

**Keywords:** personalized intervention, real-time monitoring, seizure detection, digital health, epilepsy management, wearable technology

## Abstract

This review explores recent advancements in wearable digital health technology specifically designed to manage epilepsy. Epilepsy presents unique challenges in monitoring and management due to the unpredictable nature of seizures. Wearable devices offer continuous monitoring and real-time data collection, providing insights into seizure patterns and trends. Wearable technology is important in epilepsy management because it enables early detection, prediction, and personalized intervention, empowering patients and healthcare providers. Key findings highlight the potential of wearable devices to improve seizure detection accuracy, enhance patient empowerment through real-time monitoring, and facilitate data-driven decision-making in clinical practice. However, further research is needed to validate the accuracy and reliability of these devices across diverse patient populations and clinical settings. Collaborative efforts between researchers, clinicians, technology developers, and patients are essential to drive innovation and advancements in wearable digital health technology for epilepsy management, ultimately improving outcomes and quality of life for individuals with this neurological condition.

## Introduction and background

Epilepsy is a neurological disorder characterized by recurrent seizures, which can vary in frequency and severity among individuals [1]. Managing epilepsy presents numerous challenges, including the unpredictability of seizures, the need for accurate monitoring, and the importance of timely intervention to prevent complications and improve the quality of life for patients. Traditional methods of epilepsy management, such as medication and lifestyle modifications, may not always be sufficient to effectively control seizures or provide timely assistance during episodes [2].

Wearable digital health technology has emerged as a promising tool in managing epilepsy. These devices, typically worn on the body or integrated into clothing or accessories, offer continuous monitoring and real-time data collection, enabling healthcare providers and patients better to understand seizure patterns and trends [3]. By providing early detection, prediction, and tracking of seizures, wearable devices empower individuals with epilepsy to take proactive measures and improve their management strategies. Moreover, these technologies can enhance communication between patients and healthcare providers, leading to more personalized and effective treatment approaches [4].

This review examines the latest advancements in wearable digital health technology tailored explicitly for epilepsy management. By synthesizing current research findings and technological developments, this review aims to provide insights into wearable devices' capabilities, limitations, and potential applications in epilepsy care. Through a comprehensive exploration of recent innovations, we seek to highlight the opportunities and challenges associated with integrating wearable technology into clinical practice and patient self-management strategies, ultimately contributing to the ongoing efforts to improve epilepsy care and outcomes.

## Review

Wearable digital health technology in epilepsy management

Definition and Types of Wearable Devices

**Figure 1:**
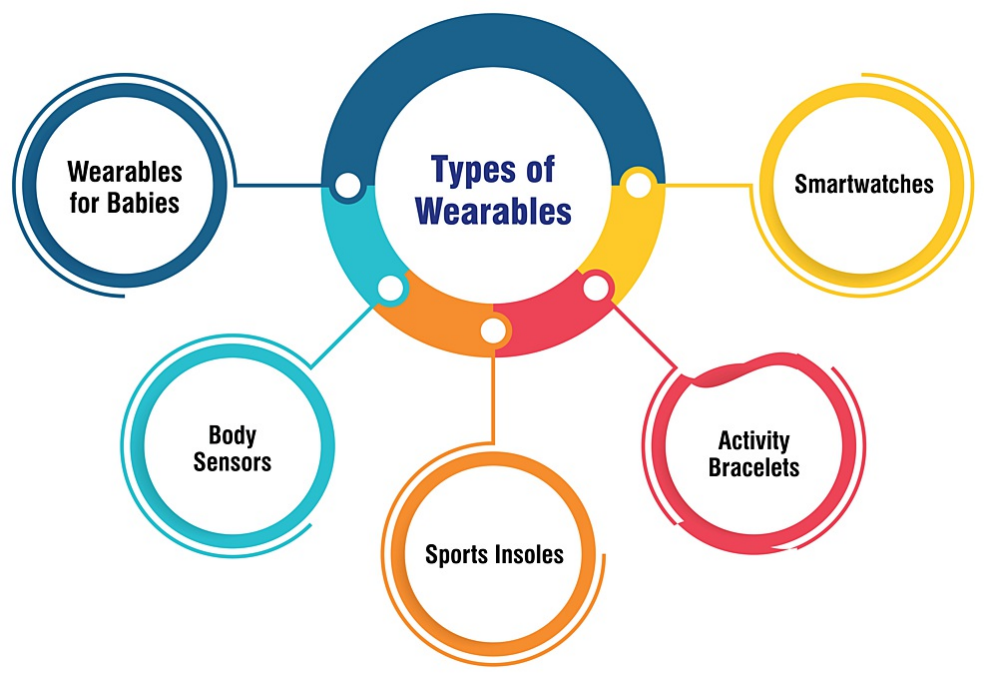
Types of wearables This figure is self-created by the corresponding author.

Wearable technology is electronic devices that track health, fitness, and other related information [5,6]. These devices, which include smartwatches, smart glasses, activity trackers, and implants, are equipped with sensors to detect, analyze, and transmit data such as vital signs, ambient information, and biofeedback [7]. They serve many purposes, from communication and entertainment to health and fitness enhancement [7]. The applications of wearable technology span various fields, such as health, medicine, fitness, aging, disability, education, transportation, enterprise, finance, gaming, and music [8]. The primary aim is to seamlessly integrate into individuals' daily lives and improve efficiency across different sectors. However, challenges such as sustaining customer engagement and addressing data security concerns are crucial for the widespread adoption of wearable technology [8]. Wearables have become integral to daily life, providing real-time data-tracking capabilities contributing to health monitoring and physical performance optimization in sports industries [9]. In addition, they offer immersive gaming experiences through devices like VR headsets and haptic technology [9]. The fashion industry has also embraced wearable technology with innovations like smart jackets that regulate body temperature based on sensors and smart rings that track steps or sleep patterns [9]. Types of wearables are shown in Figure 1.

Role of Wearable Devices in Epilepsy Monitoring and Management

Wearable devices are critical in monitoring and managing epilepsy by facilitating continuous physiological signal monitoring for seizure detection and assessing treatment response. These devices, such as wrist-worn sensors, can measure various signals, including accelerometry, electrodermal activity, photoplethysmography, and EEG, yielding high-quality data for routine use [10,11]. Wrist-worn devices have gained popularity among patients due to their convenience and effectiveness in monitoring seizure activity [11]. Advancements in wearable technology have introduced devices based on extracerebral signals, in-ear EEG devices, and ambulatory EEG systems, offering non-invasive monitoring options [11,12]. These innovations enable the development of improved prediction systems and alarm mechanisms to aid patients in predicting and managing seizures effectively [12]. Wearable devices leveraging cardiorespiratory signals have demonstrated promising results in seizure prediction, achieving high levels of sensitivity and specificity through real-time monitoring [12]. Furthermore, machine learning techniques have been integrated into wearable seizure detection systems to enhance detection quality, surpassing existing real-time systems [12]. Ethernet body-worn motion sensors have also been developed to detect motion abnormalities in epileptic patients, providing additional monitoring capabilities and alert mechanisms [12]. Wearable digital health technology has revolutionized epilepsy management by offering continuous monitoring, prediction systems, and innovative alarm mechanisms that improve patient safety and well-being while providing valuable insights into seizure prediction and management [10,13].

Key Features and Functionalities of Wearable Devices for Epilepsy

**Figure 2:**
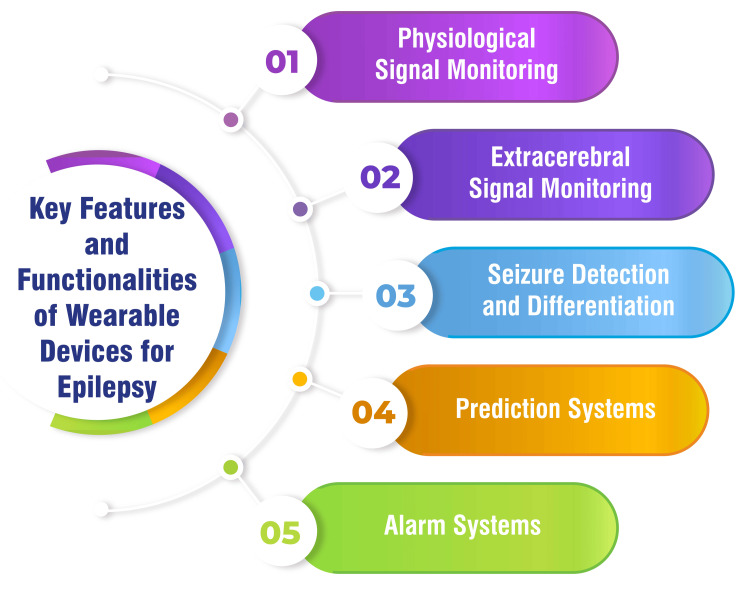
Key features and functionalities of wearable devices for epilepsy This figure is self-created by the corresponding author.

Wearable devices serve multiple functions in monitoring and managing epilepsy, leveraging various physiological signals for comprehensive assessment and intervention. These devices employ sensors to capture physiological signals such as accelerometry, electrodermal activity, photoplethysmography, and EEG, yielding high-quality data suitable for routine monitoring [11]. Innovations in wearable technology extend to extracerebral signal monitoring, exemplified by devices like in-ear EEG devices and ambulatory EEG systems, which offer non-invasive monitoring alternatives [14]. Such advancements broaden the scope of monitoring options, enhancing accessibility and comfort for patients undergoing epilepsy management. One crucial aspect of wearable technology in epilepsy management is seizure detection and differentiation, particularly in hospitals. Wearable devices are pivotal in detecting and distinguishing seizures, facilitating timely intervention, and tailored management strategies [11,14]. Moreover, wearable devices integrate prediction systems based on cardiorespiratory signals, harnessing machine learning techniques to achieve remarkable sensitivity and specificity levels [11]. These prediction systems enhance the proactive management of epilepsy by enabling early identification of potential seizure episodes. Complementing prediction and alarm systems embedded within wearable devices contributes to heightened monitoring capabilities. Ethernet body-worn motion sensors, for instance, are engineered to detect motion abnormalities in epileptic patients, furnishing an additional layer of monitoring and alerting functionalities [11]. Wearable devices represent a paradigm shift in epilepsy management, significantly advancing patient safety and well-being through real-time monitoring, predictive capabilities, and enhanced alarm mechanisms [11]. These technological innovations underscore the transformative potential of wearable digital health technology in optimizing epilepsy care. Key features and functionalities of wearable devices for epilepsy are shown in Figure 2.

Advancements in wearable digital health technology for epilepsy management

Wearable Devices for Seizure Detection

Sensors and algorithms for accurate detection: Wearable devices designed for seizure detection are significant advancements in managing epilepsy. A recent study evaluated the effectiveness of machine learning algorithms in conjunction with wearable sensors, such as body temperature, electrodermal activity, accelerometry, and photoplethysmography, in identifying seizures. Findings revealed that these algorithms could detect a wide range of seizure types more accurately than chance, offering a non-stigmatizing tool to enhance patients' overall quality of life and health outcomes [15]. These wearable digital health technologies continuously monitor and track seizure activity, furnishing valuable data for proactive epilepsy management. They employ various technologies, including EEG data, motion sensors, and heart rate monitors, to detect seizures and predict the likelihood of future episodes, thus enabling early intervention and personalized treatment plans [16]. While these devices offer benefits such as real-time alerts to patients and caregivers, reducing the risk of sudden unexpected death in epilepsy, they do have limitations. For instance, they may not detect all types of seizures or be affordable for everyone. It is essential to collaborate with healthcare providers to select the appropriate device based on factors such as seizure type detection accuracy, comfort, false alarm rates, and budget considerations [17].

Real-time monitoring and alert systems: A groundbreaking wearable device has been developed for automated real-time detection of epileptic seizures, incorporating sensors such as accelerometers, pulse oximeters, and vibration sensors to evaluate body movement, heart rate variability, oxygen saturation, and jerky movements [13]. This device aims to efficiently and economically detect epileptic seizures in real time, providing a promising solution for enhanced patient care. Wearable technology plays a vital role in seizure detection and monitoring. Devices like smartwatches are programmed to respond to repetitive shaking movements indicative of seizure activity by alerting caregivers or loved ones for prompt assistance [18]. While these devices offer advantages such as recognizing specific seizure activities characterized by significant movements, they may have limitations in detecting all seizure types and can be costly for specific individuals. Research has demonstrated that leveraging wearable sensors and machine learning algorithms can facilitate the automatic detection of various seizure types with high levels of accuracy [15]. These advancements in wearable digital health technology not only improve the quality of life for individuals with epilepsy but also contribute to reducing mortality rates associated with epileptic seizures, particularly in low-resource settings where access to expertise and treatment is limited [19].

Wearable Devices for Seizure Prediction

Machine learning and AI algorithms for predictive analytics: Advancements in wearable digital health technology for epilepsy management, particularly in seizure prediction, have marked significant progress. Researchers are harnessing machine learning algorithms and wearable devices to forecast seizures, aiming to elevate patient safety and quality of life [15,20-23]. These innovations entail the development of algorithms and wearable devices that analyze a patient's brain wave data in real-time to predict seizures, empowering individuals with epilepsy to take proactive measures such as finding a safe environment or administering medication to forestall an imminent seizure [20]. Machine learning models are being employed to enhance the precision of seizure prediction and diminish false alarms, which holds particular importance for patients with drug-resistant epilepsy [15]. Furthermore, wearable systems are being crafted to predict epileptic seizures by detecting anomalies in heart rate variability, showcasing the potential for non-invasive predictive methods that can be seamlessly integrated into smart devices and mobile phones [21]. Additionally, research has demonstrated the feasibility of utilizing deep learning models to automatically detect seizures and classify seizure types based on EEG data, laying the groundwork for more accurate and tailored seizure management strategies [15]. In summary, these advancements underscore the promising synergy between wearable digital health technology and machine learning algorithms in transforming epilepsy management and facilitating early detection, intervention, and personalized care for individuals with epilepsy.

Feedback mechanisms for personalized prediction: Wearable devices for seizure prediction have witnessed significant strides in recent years, particularly in machine learning-based anomaly detection of heart rate variability (HRV). The wearable epileptic seizure prediction system devised by Yamakawa et al. employs machine learning algorithms to scrutinize HRV indices, compute T2 and Q values, and identify anomalies preceding a seizure [21]. This system has yielded promising outcomes in predicting seizures based on HRV data. Another approach entails leveraging wearable technology to anticipate seizure likelihood by analyzing biomarkers indicative of seizure and epileptic activity cycles. For instance, heart rate and temperature may be biomarkers for seizure cycles [24]. Seizures have been observed to synchronize with underlying circadian and multiday cycles in heart rate, suggesting a propensity for seizures to occur at specific phases of individual-specific heart rate cycles [24]. In addition to HRV analysis, researchers have explored the utilization of wearable sensors and machine-learning algorithms for seizure detection [15]. These devices can identify anomalies in physiological signals, such as alterations in heart rate, skin conductance, or acceleration, signaling the onset of a seizure [12]. Overall, wearable devices hold promise in furnishing personalized prediction mechanisms for seizure detection and forecasting, thereby substantially enhancing the quality of life for individuals with epilepsy through early detection, intervention, and personalized treatment strategies [24].

Wearable Devices for Seizure Tracking and Management

Data visualization and analysis tools: The development of data visualization and analysis tools tailored for wearable technology represents a pivotal step in enhancing healthcare delivery. A notable study focused on creating CarePortal, a data analytics dashboard designed to visualize and interpret patient wearable data for clinicians [25]. Using a participatory design with clinicians, researchers crafted an interactive web application synthesizing symptomatic health data gleaned from wearable smartwatches [25]. Wearable technology endeavors to enrich lifestyles by mining physiological conditions data. Integral to this process is developing software algorithms essential for interpreting raw data from wearables, thus facilitating accurate diagnoses and furnishing personalized recommendations grounded in peer group comparisons [26]. Compelling visualizations are pivotal in unearthing pertinent health patterns from multi-sensor real-time wearable devices capturing vital data. This iterative process aids in deciphering health patterns and making informed decisions based on the amassed data [27]. Research endeavors have leveraged observational data from consumer apps and wearable devices to scrutinize human health behaviors, outlining best practices for analyzing large-scale data acquired through the routine use of commercial wearables and smartphone apps. These analyses focus on physical activity, weight, diet, sleep, blood pressure, and heart rate monitoring [28]. The amalgamation of AI, data science, and wearable devices heralds a transformative era in healthcare, automating tasks, dissecting large datasets for early disease diagnosis, fashioning personalized treatment plans, monitoring chronic conditions, and bolstering overall healthcare accessibility and efficacy [29].

Integration with electronic health records (EHRs) for comprehensive management: The integration of wearable devices with EHRs epitomizes a significant stride in healthcare technology, proffering myriad benefits. By enabling real-time synchronization between EHR systems and wearable devices, healthcare providers can aptly monitor patient health indicators, culminating in enhanced treatment outcomes [30,31]. This integration facilitates the seamless transfer of vital signs data from wearable devices into EHR systems, obviating manual data entry processes and ensuring accurate and timely access to patient health information [30]. Augmenting remote patient monitoring capabilities, the integration of wearable technology with EHR systems enables healthcare professionals to continuously monitor patients' health parameters and extend care to remote locales [32]. Furthermore, this integration streamlines telemedicine practices, furnishing physicians with a comprehensive vista of their patient's health data, thereby fostering improved care coordination and patient outcomes [32]. Wearable devices, encompassing fitness trackers, smartwatches, and assorted sensors, can seamlessly transmit data such as heart rate, activity levels, sleep patterns, and more directly to EHR systems, enriching the quantity and quality of data available to healthcare providers [32]. In essence, the fusion of wearable technology with EHR systems promotes interoperability, streamlines data collection processes, and augments patient care through remote monitoring and telemedicine initiatives [30-32]. As the wearable technology market burgeons, with an anticipated value of $60.48 billion by 2027, integration with EHR software assumes heightened significance in delivering quality care and augmenting patient outcomes [30-32].

Challenges and limitations

Accuracy and Reliability of Wearable Devices

Wearable devices offer a promising avenue for monitoring various health metrics, but their accuracy and reliability can vary across different parameters. In laboratory-based settings, these devices have demonstrated good accuracy in measuring heart rate, typically within ±3% on average [33]. Certain brands, such as Apple Watch, Fitbit, and Garmin, tend to perform well in heart rate measurement, although differences in accuracy may exist between device brands [33,34]. Regarding step count measurement, wearable devices exhibit accuracy in controlled environments, yet variations may occur depending on the brand and device type. While brands like Apple Watch and Samsung have fewer studies compared to others, they demonstrate consistent step count estimates within tight ranges [33]. However, when it comes to estimating energy expenditure, wearable devices may need more accuracy. Fitbit devices are an exception, as they tend to measure energy expenditure within acceptable limits. Nevertheless, there remains significant variability in the estimates, and accuracy can differ depending on the specific device model [33]. Regarding oxygen saturation estimation, wrist-worn activity trackers may need more accuracy for sports and healthcare applications. Although devices like the Apple Watch and Garmin smartwatches demonstrate better accuracy than other popular smartwatches, they have yet to undergo thorough validation [34]. For epilepsy management, while research on the accuracy and reliability of wearable devices is limited, it is crucial to consider the overall accuracy and reliability of these devices when evaluating their potential for monitoring and managing epileptic seizures [35]. Thus, while wearable devices offer promising capabilities across various health metrics, carefully considering their accuracy and reliability is essential for their effective use in healthcare applications.

User Acceptance and Adherence

Wearable technology confronts several challenges concerning user acceptance, data security, and ethical considerations, necessitating careful address for successful implementation [36]. Research indicates that individuals engaging with wearable devices, such as smartwatches for medication reminders, exhibit improved adherence to treatment regimens [36]. Chronic diseases significantly impact the acceptance and apprehensions of adopting innovative health monitoring sensors [37]. Therefore, understanding these factors is paramount in devising effective interventions tailored to meet specific patient requirements. Usability among older adults remains a crucial consideration in the integration of wearable sensors for health monitoring [38]. Comparative research on usability highlighted the importance of factoring individual and environmental variables in device selection to foster long-term compliance. Factors such as comfort, discretion, and the burden of charging play pivotal roles in user acceptance and adherence. In Parkinson's disease management, a study on a digital health technology system aimed at monitoring mobility and evaluating medication adherence showcased high adherence rates among participants, underscoring the feasibility and usability of such systems [39]. Furthermore, the ownership of smart devices is intertwined with demographic factors, including gender, age, education, and employment status, influencing the acceptability of sharing digital health data [40]. An in-depth comprehension of these factors is instrumental in optimizing the design of research studies and clinical trials employing smart devices.

Data Privacy and Security Concerns

Wearable devices are pivotal in tracking and monitoring personal health data, including heart rate, activity levels, and sleep patterns, with the collected information typically stored in the cloud. However, concerns arise regarding third-party access to this data, raising privacy and security issues, as the data may be utilized for various purposes without explicit consent from users [41,42]. Existing regulatory frameworks such as the General Data Protection Regulation (GDPR) and the Health Insurance Portability and Accountability Act (HIPAA) offer some protection for personal health data. Nonetheless, a pressing need remains for more robust regulations and industry self-regulation to ensure the appropriate use and safeguarding of this sensitive information [41,42]. Privacy breaches and data misuse have been documented, underscoring the importance of informed consent and user autonomy in data collection and usage. High-profile cases such as Strava inadvertently disclosing sensitive locations and Fitbit facing legal action for selling personal health data highlight the risks associated with inadequate privacy measures [41,42]. Maintaining user awareness and obtaining explicit consent for data sharing are essential components in preserving privacy and mitigating the potential misuse of personal health information [42]. It is imperative to ensure that users are well-informed about how their data is utilized to foster transparency and trust in wearable technology. Implementing robust data security measures, including encryption protocols and access controls, is crucial for safeguarding sensitive health data stored by wearable devices. These security measures can help mitigate the risks of data breaches and unauthorized access, thus bolstering user trust and confidence in wearable technology [42].

Regulatory and Reimbursement Issues

The reimbursement framework for AI/ML technologies within healthcare is evolving, presenting limited opportunities for direct reimbursement. Challenges arise due to the conventional emphasis on clinicians as the primary providers of healthcare services, which can impede the integration of AI/ML technologies into routine healthcare practices [43]. Regulatory compliance is a critical aspect governed by the FDA in overseeing AI/ML technologies intended for healthcare use. The FDA's scope of oversight concerning AI/ML software is expanding, with an increasing number of devices incorporating AI/ML functionalities undergoing review and authorization through various FDA processes such as 510(k) clearance, de novo submissions, and premarket approval (PMA) [43]. The utilization of AI/ML in healthcare settings intersects with state regulations governing medical practice and licensed professionals, thereby significantly influencing the adoption and implementation of these technologies. Compliance with local regulations is paramount and necessitates careful consideration to ensure alignment with state laws [43]. The dynamic landscape of digital health technologies introduces novel challenges for regulators, particularly concerning adaptability and product innovation. Addressing the regulatory gap in digital health requires alternative pathways that balance effective regulation with the promotion of innovation [44]. Interoperability between wearable devices and existing healthcare systems is imperative to facilitate seamless data sharing and integration into clinical workflows. Establishing standardized protocols for data communication is essential to mitigate data silos and operational inefficiencies [44].

Future directions and opportunities

Integration of Multiple Sensors and Modalities for Enhanced Accuracy

Integrating multiple sensors and modalities in wearable technology is bolstering accuracy and broadening the capabilities of health monitoring devices. Wearable sensors utilize a range of physical, chemical, and biological sensors to extract real-time physiological information, non-invasive or minimally invasive [45]. These sensors can be integrated into wearable forms such as glasses, jewelry, wristwatches, fitness bands, and textiles, facilitating early detection and monitoring of conditions like COVID-19 and Parkinson's disease through biophysical signals [45]. Wearable sensors encompass mechanical, electrical, optical, and chemical modalities, each presenting unique challenges and opportunities for advancement [46]. While mechanical, electrical, and optical sensors have made significant strides in miniaturization and flexibility, chemical sensing modalities encounter hurdles in commercial adoption, particularly for non-invasive applications [46]. The future trajectory of wearable sensors lies in integrating hybrid mechanisms to comprehensively monitor various physiological parameters [47]. This integration entails merging flexible power units with wearable sensors to create self-powered systems capable of continuous monitoring [47]. The forefront of wearable sensor technology delves into precision medicine and personalized healthcare, where wearable devices are pivotal in furnishing accurate and continuous health data for enhanced medical diagnosis and monitoring [47]. Future trends encompass integrating additional mechanisms into single devices to augment sensing capabilities, refining materials and structures for heightened sensitivity, and pioneering novel sensor technologies for personalized healthcare applications [47,48]. Wearable sensors are poised to revolutionize healthcare by providing advanced monitoring capabilities tailored to individual health needs, fostering innovations in remote patient monitoring and decentralized healthcare systems.

Development of Closed-Loop Systems for Personalized Intervention

Developing closed-loop systems for personalized intervention in healthcare stands at the forefront of cutting-edge research, holding immense promise for improving patient outcomes and treatment efficacy. These systems harness wearable technology and real-time monitoring to deliver integrated and personalized treatment solutions, empowering patients to monitor, track, and enhance their health [49]. By leveraging closed-loop systems, healthcare providers can administer interventions tailored to individual needs, fostering more effective and targeted care [50]. Research endeavors in this domain are concentrated on crafting personalized closed-loop controllers for diverse medical applications, such as managing medically induced comas in intensive care units [50]. These systems monitor inter and intra-subject variabilities in the brain's response to treatments like anesthetic infusion rates, facilitating precise control and optimizing therapy delivery [50]. Through the integration of real-time tracking of these variabilities, closed-loop systems can heighten control precision, enhance clinical feasibility, and minimize interruptions in therapy administration [50]. The realm of closed-loop systems transcends traditional medical interventions to encompass brain stimulation for mental disorders and bioenergy-based treatments for integrated medical care [51]. These innovative approaches open new avenues for personalized and more effective interventions, laying the groundwork for intelligent and integrated medical systems tailored to individual patient needs [51]. Overall, the advancement of closed-loop systems heralds a significant stride in healthcare technology, offering personalized solutions that elevate patient care and treatment outcomes.

Collaboration Between Technology Developers, Clinicians, and Patients

Collaboration among technology developers, clinicians, and patients is pivotal in unlocking the full potential of wearable digital health technology and ushering in personalized and revolutionary medical advancements. By joining forces, technologists, data scientists, and clinicians can integrate wearable technology into healthcare, focusing on personalized health management beyond traditional healthcare settings [41]. This collaborative effort entails harnessing wearable devices for real-time monitoring of physiological parameters, empowering individuals to take proactive steps toward managing their health and well-being [52]. Samsung's initiatives are prime examples of successful collaborations in the digital health arena, as the company partners with universities and academic hospitals to spearhead innovative wellness approaches through wearables [53]. These collaborative endeavors aim to transform the digital health landscape by exploring solutions such as wearable sleep-tracking devices to enhance sleep quality, quantifying resilience and frailty using biometric data collected by devices like the Galaxy Watch and addressing cardiovascular disease through groundbreaking sensor technologies [53]. In navigating challenges and charting a course for wearable devices in digital health, emphasis is placed on data quality, interoperability, health equity, and fairness in wearables' application for healthcare monitoring, screening, detection, and prediction [54]. To propel the field forward effectively, recommendations include establishing local standards of quality, ensuring the interoperability of devices, advocating for universal access to wearable technology, and prioritizing representativity in data collection to foster fair and equitable healthcare practices [54]. Through collaborative efforts and concerted actions, stakeholders can pave the way for a future where wearable digital health technology optimally serves individuals' diverse healthcare needs.

Addressing Disparities in Access to Wearable Technology

Addressing disparities in access to wearable technology is paramount for ensuring equitable healthcare outcomes. Studies have shed light on significant inequities in the utilization of wearable health devices, with factors such as age, education, and income exerting influence on their adoption among individuals with cardiovascular disease or those at risk for it [55]. To narrow this gap, concerted efforts are necessary to enhance access and position wearables as indispensable health tools to improve health outcomes and mitigate disparities [55]. Cost and education emerge as pivotal factors shaping access to wearables, emphasizing the imperative to bolster awareness and affordability of these devices in marginalized communities [56]. Reconfiguring processes within regulatory bodies like the FDA to incorporate racial equity considerations during device approval can help rectify deficits in representation and ensure accurate functionality across diverse populations [56]. Moreover, initiatives to augment research funding for developing inclusive devices and interventions to mitigate disparities in telehealth usage are indispensable in advancing equitable access to digital health technologies [56]. Experts caution that access to wearables could evolve into a social determinant of health, underscoring the urgency of addressing barriers such as cost, awareness, and language to facilitate broad community participation in digital health initiatives [57]. Strategies encompassing educational outreach, public or private financial investments, and cultural sensitivity play a pivotal role in fostering the widespread adoption of digital health devices across diverse populations [57]. Through collaborative efforts and targeted interventions, stakeholders can work toward dismantling barriers and ensuring all individuals have equal access to the transformative benefits of wearable technology in healthcare.

## Conclusions

In conclusion, this review has illuminated the pivotal role of wearable digital health technology in managing epilepsy, addressing the challenges inherent in traditional approaches while presenting new opportunities for personalised care. By harnessing continuous monitoring and real-time data collection, these devices offer invaluable insights into seizure patterns and trends, empowering patients and healthcare providers to make informed decisions and take proactive measures. The implications for clinical practice are profound, as wearable technology enables more comprehensive and timely interventions, potentially leading to improved outcomes and quality of life for individuals with epilepsy. However, to fully realise the potential of wearable devices in epilepsy management, further research is warranted. Validation studies, longitudinal research, user-centred design, interoperability enhancements, and regulatory considerations are all critical areas for future exploration and development. Through collaborative efforts and a commitment to innovation, we can advance the wearable digital health technology field and continue to enhance epilepsy care, ultimately benefiting patients and healthcare systems alike.

## References

[1] (2024). Epilepsy - Seizure types, symptoms and treatment options. https://www.aans.org/Patients/Neurosurgical-Conditions-and-Treatments/Epilepsy.

[2] (2024). Epilepsy and seizures. https://www.ninds.nih.gov/health-information/disorders/epilepsy-and-seizures.

[3] Lu L, Zhang J, Xie Y, Gao F, Xu S, Wu X, Ye Z (2020). Wearable health devices in health care: narrative systematic review. JMIR Mhealth Uhealth.

[4] Masoumian Hosseini M, Masoumian Hosseini ST, Qayumi K, Hosseinzadeh S, Sajadi Tabar SS (2023). Smartwatches in healthcare medicine: assistance and monitoring; a scoping review. BMC Med Inform Decis Mak.

[5] (2024). What is wearable technology - Definition, meaning and examples. https://www.investopedia.com/terms/w/wearable-technology.asp.

[6] (2024). Definition, types & history. https://blogs.baylor.edu/edc5370/wearable-tech/definition-types-and-history/.

[7] (2024). What is wearable technology (wearables)? Definition and examples. https://www.investopedia.com/terms/w/wearable-technology.asp.

[8] (2024). Wearables, wearable technology & devices. https://www.happiestminds.com/insights/wearable-technology/.

[9] (2024). What is wearable technology? examples of wearables. https://builtin.com/wearables.

[10] Feb 22;390736-745 TNEJ of MWDHT for ENEJM 2024, Donner E, Devinsky O, MEDLINE®/PubMed® DFF (2024). Wearable digital health technology for epilepsy. https://www.practiceupdate.com/content/wearable-digital-health-technology-for-epilepsy/162815.

[11] Ong JS, Wong SN, Arulsamy A, Watterson JL, Shaikh MF (2022). Medical technology: a systematic review on medical devices utilized for epilepsy prediction and management. Curr Neuropharmacol.

[12] Li W, Wang G, Lei X, Sheng D, Yu T, Wang G (2022). Seizure detection based on wearable devices: a review of device, mechanism, and algorithm. Acta Neurol Scand.

[13] Habtamu M, Tolosa K, Abera K (2023). A novel wearable device for automated real-time detection of epileptic seizures. BMC Biomed Eng.

[14] Johansson D, Malmgren K, Alt Murphy M (2018). Wearable sensors for clinical applications in epilepsy, Parkinson's disease, and stroke: a mixed-methods systematic review. J Neurol.

[15] Tang J, El Atrache R, Yu S (2021). Seizure detection using wearable sensors and machine learning: setting a benchmark. Epilepsia.

[16] Amadi A (2024). Harnessing the power of wearable digital health devices in epilepsy care. https://medriva.com/health/digital-health/harnessing-the-power-of-wearable-digital-health-devices-in-epilepsy-care/.

[17] Contributors WE (2024). What to know about seizure alert devices. https://www.webmd.com/epilepsy/what-to-know-seizure-alert-devices.

[18] (2024). Seizure monitors & devices. https://epilepsyfoundation.org.au/understanding-epilepsy/epilepsy-and-seizure-management-tools/seizure-monitors-devices/.

[19] Rukasha T, I Woolley S, Kyriacou T, Collins T (2020). Evaluation of wearable electronics for epilepsy: a systematic review. Electronics.

[20] (2024). Wearable devices could help predict seizures. https://news.fiu.edu/2022/seizure-prediction-fiu-researchers-work-to-improve-lives-for-epileptics.

[21] Yamakawa T, Miyajima M, Fujiwara K (2020). Wearable epileptic seizure prediction system with machine-learning-based anomaly detection of heart rate variability. Sensors (Basel).

[22] Nasseri M, Pal Attia T, Joseph B (2021). Ambulatory seizure forecasting with a wrist-worn device using long-short term memory deep learning. Sci Rep.

[23] Halimeh M, Jackson M, Vieluf S, Loddenkemper T, Meisel C (2023). Explainable AI for wearable seizure logging: impact of data quality, patient age, and antiseizure medication on performance. Seizure.

[24] Stirling RE, Grayden DB, D'Souza W (2021). Forecasting seizure likelihood with wearable technology. Front Neurol.

[25] Sadhu S, Solanki D, Brick LA, Nugent NR, Mankodiya K (2023). Designing a clinician-centered wearable data dashboard (CarePortal): participatory design study. JMIR Form Res.

[26] Angelides MC, Wilson LA, Echeverría PL (2018). Wearable data analysis, visualisation and recommendations on the go using android middleware. Multimed Tools Appl.

[27] Suter S, Spinner G, Hoelz B, Rey S, Thanabalasingam S, Eckstein J, Hirsch S (2022). Visualization and analysis of wearable health data from COVID-19 patient. Publikationen Life Sciences und Facility Management.

[28] Hicks JL, Althoff T, Sosic R (2019). Best practices for analyzing large-scale health data from wearables and smartphone apps. NPJ Digit Med.

[29] Bajwa J, Munir U, Nori A, Williams B (2021). Artificial intelligence in healthcare: transforming the practice of medicine. Future Healthc J.

[30] (2024). Integration of wearable devices with EHR. https://www.apexon.com/blog/integration-of-wearable-devices-with-ehr/.

[31] (2024). EHR integration with wearable devices. SyS Creat. - IT Manag. Compliance Consult. Co. Can.

[32] (2024). Telemedicine and EHR integration with wearable technology. PrognoCIS EHR.

[33] Fuller D, Colwell E, Low J (2020). Reliability and validity of commercially available wearable devices for measuring steps, energy expenditure, and heart rate: systematic review. JMIR Mhealth Uhealth.

[34] Shei RJ, Holder IG, Oumsang AS, Paris BA, Paris HL (2022). Wearable activity trackers-advanced technology or advanced marketing?. Eur J Appl Physiol.

[35] Brinkmann BH, Karoly PJ, Nurse ES (2021). Seizure diaries and forecasting with wearables: epilepsy monitoring outside the clinic. Front Neurol.

[36] Ph.D DA: Wearable Technology (2024). Wearable technology: Innovation, adherence, and clinical outcomes. https://www.pharmasalmanac.com/articles/wearable-technology-innovation-adherence-and-clinical-outcomes.

[37] Materia FT, Smyth JM (2024). Acceptability and concerns about innovative wearable health sensors in persons with and without chronic disease diagnosis. Internet Interv.

[38] Keogh A, Dorn JF, Walsh L, Calvo F, Caulfield B (2020). Comparing the usability and acceptability of wearable sensors among older Irish adults in a real-world context: observational study. JMIR Mhealth Uhealth.

[39] Debelle H, Packer E, Beales E (2023). Feasibility and usability of a digital health technology system to monitor mobility and assess medication adherence in mild-to-moderate Parkinson's disease. Front Neurol.

[40] Shandhi MM, Singh K, Janson N (2024). Assessment of ownership of smart devices and the acceptability of digital health data sharing. NPJ Digit Med.

[41] Silva JP da: Privacy Data (2024). Ethics of wearable digital health technology. Digit. Health Med.

[42] jirehl jirehl (2024). Privacy, data security concerns rise as healthcare wearables gain popularity. https://thehealthcaretechnologyreport.com/privacy-data-security-concerns-rise-as-healthcare-wearables-gain-popularity/.

[43] (2024). AI in medical devices and healthcare: Opportunities, challenges, and what lies ahead. https://www.morganlewis.com/pubs/2023/03/ai-in-medical-devices-and-healthcare-opportunities-challenges-and-what-lies-ahead.

[44] Iqbal JD, Biller-Andorno N (2022). The regulatory gap in digital health and alternative pathways to bridge it. Health Policy Technol.

[45] Ates HC, Nguyen PQ, Gonzalez-Macia L, Morales-Narváez E, Güder F, Collins JJ, Dincer C (2022). End-to-end design of wearable sensors. Nat Rev Mater.

[46] Heikenfeld J, Jajack A, Rogers J (2018). Wearable sensors: modalities, challenges, and prospects. Lab Chip.

[47] Zeng X, Deng HT, Wen DL, Li YY, Xu L, Zhang XS (2022). Wearable multi-functional sensing technology for healthcare smart detection. Micromachines (Basel).

[48] (2024). Upcoming trends in wearable healthcare monitoring technology. https://www.mddionline.com/digital-health/upcoming-trends-in-wearable-healthcare-monitoring-technology.

[49] Johnson KB, Wei WQ, Weeraratne D (2021). Precision medicine, AI, and the future of personalized health care. Clin Transl Sci.

[50] Yang Y, Lee JT, Guidera JA (2019). Developing a personalized closed-loop controller of medically-induced coma in a rodent model. J Neural Eng.

[51] Zhang G, Chen Y, Zhou W, Chen C, Liu Y (2023). Bioenergy-based closed-loop medical systems for the integration of treatment, monitoring, and feedback. Small Sci.

[52] Wall C, Hetherington V, Godfrey A (2023). Beyond the clinic: the rise of wearables and smartphones in decentralising healthcare. NPJ Digit Med.

[53] (2024). Samsung explores potential of wearables in digital health collaborations. https://www.pymnts.com/healthcare/2023/samsung-explores-potential-wearables-digital-health-collaborations/.

[54] Canali S, Schiaffonati V, Aliverti A (2022). Challenges and recommendations for wearable devices in digital health: data quality, interoperability, health equity, fairness. PLOS Digit Health.

[55] (2024). Study finds people who need wearable health devices the most use them the least. https://newsroom.heart.org/news/study-finds-people-who-need-wearable-health-devices-the-most-use-them-the-least..

[56] Raza MM, Venkatesh KP, Kvedar JC (2023). Promoting racial equity in digital health: applying a cross-disciplinary equity framework. NPJ Digit Med.

[57] (2024). Access to wearables could become a social determinant of health, researchers warn. https://www.healthcareitnews.com/news/access-wearables-could-become-social-determinant-health-researchers-warn.

